# Increased serum levels of fractalkine and mobilisation of CD34^+^CD45^−^ endothelial progenitor cells in systemic sclerosis

**DOI:** 10.1186/s13075-017-1271-7

**Published:** 2017-03-20

**Authors:** Audrey Benyamine, Jérémy Magalon, Sylvie Cointe, Romaric Lacroix, Laurent Arnaud, Nathalie Bardin, Pascal Rossi, Yves Francès, Fanny Bernard-Guervilly, Gilles Kaplanski, Jean-Robert Harlé, Pierre-Jean Weiller, Philippe Berbis, David Braunstein, Elisabeth Jouve, Nathalie Lesavre, Françoise Couranjou, Françoise Dignat-George, Florence Sabatier, Pascale Paul, Brigitte Granel

**Affiliations:** 1 Internal Medicine Department, Assistance Publique-Hôpitaux de Marseille (APHM), CHU Nord, 13015 Marseilles, France; 2 Haematology and Vascular Biology Laboratory, APHM, CHU Conception, 13005 Marseilles, France; 3 Vascular Research Centre of Marseille (VRCM) UMR-S1076, INSERM, Aix-Marseille Université, 13385 Marseilles, Cedex, France; 4 Culture and Cell Therapy Laboratory, CICBT 1409, APHM, CHU Conception, 13005 Marseilles, France; 5 Immunology Laboratory, APHM, CHU Conception, 13005 Marseilles, France; 6 Internal Medicine Department, APHM, CHU Conception, 13005 Marseilles, France; 7 Internal Medicine Department, APHM, CHU Timone, 13005 Marseilles, France; 8 Dermatology Department, APHM, CHU Nord, 13015 Marseilles, France; 9 Centre d’Investigation Clinique Centre de Pharmacologie Clinique et d’Evaluations Thérapeutiques, APHM, CHU Timone, CHU Nord, Marseille, France

**Keywords:** Systemic sclerosis, Fractalkine, Endothelial progenitor cells, Endothelial microparticles, Circulating endothelial cells, Vascular endothelial growth factor, Endothelin-1, Endothelial homeostasis

## Abstract

**Background:**

The disruption of endothelial homeostasis is a major determinant in the pathogenesis of systemic sclerosis (SSc) and is reflected by soluble and cellular markers of activation, injury and repair. We aimed to provide a combined assessment of endothelial markers to delineate specific profiles associated with SSc disease and its severity.

**Methods:**

We conducted an observational, single-centre study comprising 45 patients with SSc and 41 healthy control subjects. Flow cytometry was used to quantify circulating endothelial microparticles (EMPs) and CD34^+^ progenitor cell subsets. Colony-forming unit-endothelial cells (CFU-ECs) were counted by culture assay. Circulating endothelial cells were enumerated using anti-CD146-based immunomagnetic separation. Blood levels of endothelin-1, vascular endothelial growth factor (VEGF) and soluble fractalkine (s-Fractalkine) were evaluated by enzyme-linked immunosorbent assay. Disease-associated markers were identified using univariate, correlation and multivariate analyses.

**Results:**

Enhanced numbers of EMPs, CFU-ECs and non-haematopoietic CD34^+^CD45^−^ endothelial progenitor cells (EPCs) were observed in patients with SSc. Patients with SSc also displayed higher serum levels of VEGF, endothelin-1 and s-Fractalkine. s-Fractalkine levels positively correlated with CD34^+^CD45^−^ EPC numbers. EMPs, s-Fractalkine and endothelin-1 were independent factors associated with SSc. Patients with high CD34^+^CD45^−^ EPC numbers had lower forced vital capacity values. Elevated s-Fractalkine levels were associated with disease severity, a higher frequency of pulmonary fibrosis and altered carbon monoxide diffusion.

**Conclusions:**

This study identifies the mobilisation of CD34^+^CD45^−^ EPCs and high levels of s-Fractalkine as specific features of SSc-associated vascular activation and disease severity. This signature may provide novel insights linking endothelial inflammation and defective repair processes in the pathogenesis of SSc.

**Electronic supplementary material:**

The online version of this article (doi:10.1186/s13075-017-1271-7) contains supplementary material, which is available to authorized users.

## Background

Systemic sclerosis (SSc) is a systemic autoimmune disease characterised by vascular damage and fibrosis [1]. The blood vessel is the primary target for both initiating and propagating the local immune activation and fibrosis. This vascular hypothesis has been underscored by the impairment of the flow-mediated dilation (FMD) of the brachial artery, a validated, non-invasive physiological measure of endothelial function [2].

The disruption of endothelial integrity involves an altered balance between lesion and repair processes that can be assessed by non-invasive endothelium-derived biomarkers which our group has contributed to identify and standardise [3, 4]. These markers include circulating endothelial cells (CECs) that enter the bloodstream following detachment of stressed endothelial cells from the vessel wall and endothelial microparticles (EMPs) that are shed during the membrane remodelling of activated or apoptotic endothelial cells. These markers have been shown to have diagnostic and prognostic value in cardiovascular diseases [4–6], but their significance in inflammatory and autoimmune diseases is less well established [7–9].

In response to vascular injury, endothelial repair mechanisms have been shown to involve the recruitment of progenitor cells (PCs) [10]. In their pioneering work, Asahara et al. [10] identified a bone marrow-derived circulating population of PCs expressing CD34 and kinase insert domain receptor (KDR, vascular endothelial growth factor receptor 2 [VEGFR-2]) antigens that displays the capacity to differentiate into an endothelial phenotype and contribute to physiological or pathological neovascularisation. These cells were referred to as *endothelial progenitor cells* (EPCs).

From that time, extensive research has led to the recognition that EPCs represent a highly heterogeneous cell compartment. Indeed, various circulating subpopulations with different stages of maturation, lineage origin and functional properties contribute to the “EPC pool” [11, 12]. Following the first report of decreased levels of circulating EPCs in SSc [13], several controversial studies have addressed their quantitative and functional alterations [14–23]. These discrepancies may arise from the clinical characteristics of the enrolled patients with SSc and the disparate methodologies used to analyse EPCs. Indeed, despite the effort to find a consensus [22], these methods based on flow cytometric analyses or on ex vivo culture protocols have sometimes led to the assessment of distinct cell populations.

Importantly, most of the literature in the SSc field has focused on cells that belong to the haematopoietic lineage [23]. Indeed, recent clarifications in EPC identity indicate that a combination of CD34, CD133 and KDR markers enumerate mostly bone marrow-derived haematopoietic cells or progenitors that correlate with vascular endothelial status [11]. These cells are now designated as circulating angiogenic cells (CACs) to reflect their potential to sustain angiogenesis but lack de novo vessel-forming activity [24]. Additionally, the colony-forming unit-endothelial cell (CFU-EC) assay introduced by Hill et al. allowed for the description of the CFU-ECs as relevant biomarkers of cardiovascular risk [25]. Increased CFU-EC formation was also associated with the inflammatory response to endothelial injury [26]. These CFU-ECs exhibit characteristics of monocytes/macrophages and contribute to a paracrine support of endothelial lining repair [26].

By contrast, “true EPCs” have been identified within the CD45^−^ non-haematopoietic fraction of the CD34^+^ circulating PCs and are capable of forming highly proliferative late-outgrowth endothelial colonies. These cells, also named *endothelial colony-forming cells* (ECFCs), behave as angioblasts with a specific ability to achieve endothelial differentiation and contribute to de novo vessel formation [24]. They are unlikely derived from bone marrow, but rather belong to a pool of vascular wall-resident precursors [11]. Owing to the extreme scarcity of EPCs in peripheral blood, very few clinical studies have tackled this cell population. These investigations were restricted mainly to the cardiovascular field and suggested the potential relevance of CD34^+^CD45^−^ EPC quantification as a reflection of inflammatory [27] or mechanical vascular injury [28]. To our knowledge, this CD34^+^CD45^−^ EPC subset has never been investigated in patients with SSc.

In addition, several soluble inflammatory endothelial mediators, such as endothelin-1 [29] and soluble fractalkine (s-Fractalkine) [30], have been associated with SSc pathogenesis. Elevated endothelin-1 levels were shown to induce endothelial cell activation, fibroblast differentiation and vascular remodelling [29]. This discovery allowed for the therapeutic targeting of the endothelin-1 pathway, which led to a real clinical benefit in patients with pulmonary arterial hypertension [31] and digital ulcers [32]. Fractalkine (chemokine [C-X3-C motif] ligand 1 [CX3CL1]) is an endothelial membrane-bound adhesion molecule and a soluble chemokine after metalloprotease cleavage [33]. Increased endothelial cell surface expression and circulating s-Fractalkine levels have been described in inflammatory contexts of vascular injury, such as atherosclerosis [34] and immune diseases, including SSc [30]. The upregulation of fractalkine on activated endothelial cells allows for the recruitment and activation of immune cells expressing chemokine (C-X3-C motif) receptor 1 (CX3CR1) [35]. Polymorphisms of CX3CR1 have been associated with SSc-associated pulmonary arterial hypertension [36]. Accordingly, the disruption of the interaction between fractalkine and CX3CR1 has been shown to dampen the fibrotic process in a murine model of cytokine-induced SSc [37].

For this reason, we sought to provide an integrative view of the endothelial status based on the combined assessment of circulating biomarkers of endothelial inflammation, injury and repair. Our objective was to delineate specific profiles associated with SSc disease and severity.

## Methods

### Patients

Forty-five consecutive, unselected patients with SSc were recruited at the Department of Internal Medicine, Assistance Publique-Hôpitaux de Marseille (APHM). All the enrolled patients had a score ≥9 for SSc according to the 2013 American College of Rheumatology/European League Against Rheumatism criteria [38]. Among the patients were 44 women and 1 man with a median age of 60.38 years (IQR 51.45–72.38 years) (Table 1). Healthy volunteers (*n* = 41) were recruited for the study by the Centre d’Investigation Clinique Marseille, APHM, and used as control subjects. The control group consisted of 38 women and 3 men with a median age of 55.97 years (IQR 53.56–59.64 years) (Table 1). Written informed consent was obtained according to the Declaration of Helsinki, and the study was approved by the local ethics committee review board of Marseilles.

**Table 1:** Demographic characteristics and cardiovascular risk factors of the subjects

	Patients with SSc (*n* = 45)	Control subjects (*n* = 41)	*P* value
Sex, male/female, *n*	1/44	3/38	0.3437
Age, years, median [IQR]	61.49 (±11.95) 60.4 [51.5–72.4]	56.09 (±7.82) 56 [53.6–59.6]	0.0375
BMI, kg/m^2^, median [IQR]	23.05 (±4.63) 22.5 [20.2–23.8]	25.3 (±3.75) 24.9 [22.8–28.3]	0.0022
Systolic blood pressure, mmHg	119.11 (±16.95) 120 [105–130.25]	127.7 (±14.1) 126 [119.25–135.25]	0.0141
Diastolic blood pressure, mmHg	71.86 (±8.91) 72.5 [67.75–78.25]	79.15 (±9.71) 79 [73–84.25]	0.0006
Resting heart rate, beats/minute	71.25 (±10.24) 70.5 [64–76.25]	68.95 (±9.93) 70 [62.75–74]	0.3001
LDL cholesterol, g/L	3.29 (±1.32) 3.2 [2.36–4.28]	3.69 (±0.79) 3.59 [3.17–4.13]	0.0978
HDL cholesterol, g/L	1.72 (±0.43) 1.71 [1.4–1.95]	1.59 (±0.43) 1.51 [1.34–1.85]	0.1848
Triglycerides, g/L	1.14 (±0.47) 0.93 [0.81–1.48]	1.4 (±0.74) 1.25 [0.88–1.66]	0.1303
Carotid-radial pulse wave velocity, m/second	11.05 (±3.32) 10.5 [8.16–13]	10.11 (±3.01) 9.46 [7.62–12.05]	0.1855
Flow-mediated dilation, %	9.87 (±6.97) 9.3 [5.8–14.76]	15.29 (±8.75) 12.35 [9.09–21.38]	0.0084

*Abbreviations: BMI* Body mass index, *HDL* High-density lipoprotein, *LDL* Low-density lipoprotein, *SSc* Systemic sclerosis

Variables are described using mean (±SD) and median [IQR]

### Clinical and standard biological assessment

All subjects had a physical examination and underwent laboratory tests. Characteristics of the study population are summarised in Table 1. Arterial stiffness was assessed by measuring the right carotid-radial pulse wave velocity (PWV) using a validated, non-invasive, automated method. The FMD of the brachial artery was measured as previously reported [2].

Patients with SSc were classified as having limited cutaneous SSc or diffuse cutaneous SSc according to the criteria established by van den Hoogen et al. [38]*.* The disease duration, the presence of Raynaud’s phenomenon, pitting scars, digital ulcers, digital gangrene or telangiectasia were recorded. The modified Rodnan skin score (mRSS) was graded on a scale of 0–3 with a maximum total score of 51. Disease severity was measured on a scale of 0–4 according to the Medsger severity scale [39]. Two groups of patients were segregated on the basis of severity state: Group 1 comprised grades 0, 1 and 2, and group 2 consisted of grades 3 and 4 (Table 2).

**Table 2:** Clinical characteristics of patients with systemic sclerosis

	Patients with SSc (*n* = 45)
Diffuse/limited cutaneous subtype	16 (35.6%)/29 (64.4%)
Anti-centromere/anti-topoisomerase I	21 (46.7%)/19 (42.2%)
Disease duration ≤3 years/˃3 years	15 (34.1%)/29 (65.9%)
Medsger severity scale	
Grades 0, 1 and 2	1 (2.2%), 14 (31.1%) and 7 (15.6%)
Grades 3 and 4	22 (48.9%) and 1 (2.2%)
Pitting scars	24 (53.3%)
Digital ulcers	18 (40.0%)
Digital gangrene	4 (8.9%)
Telangiectasia	29 (64.4%)
Pulmonary function tests	
DLCO, %	97.3 [88.8–103.5]
DLCO/VA, %	67.4 [61.35–78.2]
FVC, %	99.3 [84.1–115.6]
Pulmonary fibrosis	7 (18.9%)
Systolic pulmonary arterial pressure^a^	33 [27.8–37.5]

*DLCO* Diffusing capacity of the lung for carbon monoxide, FVC Forced vital capacity, VA alveolar volume

Qualitative variables are described using counts and percentages. Quantitative variables are described using median [first quartile–third quartile]

^a^Transthoracic echocardiography assessment

Concomitant treatments of patients with SSc are described in Additional file 1: Table S1. Biological data comprised white blood cell count, haemoglobin rate, platelets count, low-density lipoprotein cholesterol, high-density lipoprotein cholesterol, triglycerides, creatinine level and C-reactive protein (CRP) level (Additional file 1: Table S2).Pulmonary involvement was determined by pulmonary function tests, including forced vital capacity (FVC), diffusing capacity of the lung for carbon monoxide (DLCO) and DLCO divided by alveolar volume (DLCO/VA). Fibrosis was diagnosed on the basis of chest computed tomography, with qualitative criteria consisting of the presence of honeycombing, ground-glass opacities, reticular abnormalities, traction bronchiectasis and septal thickening. Systolic pulmonary arterial pressure was measured by transthoracic echocardiography, and pulmonary hypertension was confirmed by right heart catheterisation. Anti-nuclear antibodies were assessed by indirect immunofluorescence analysis of HEp-2 cells. Anti-centromere and anti-topoisomerase I antibodies were measured by enzyme-linked immunosorbent assay (ELISA).

### Flow cytometric enumeration of endothelial microparticles

Control and patient samples for EMP analysis were collected and processed according to the current International Society on Thrombosis and Haemostasis guidelines [40]. Briefly, blood samples collected into 5-ml BD Vacutainer tubes containing 0.129 mol/L sodium citrate (BD Diagnostics, Franklin Lakes, NJ, USA) were subjected to two successive centrifugations (2500 × *g* for 15 minutes at room temperature), and platelet-free plasma (PFP) was prepared. The PFP was homogenised before being aliquoted and stored at −80 °C until use. Annexin V-fluorescein isothiocyanate (FITC) and the following fluorescent antibody reagents were procured from Beckman Coulter (Villepinte, France): CD31-phycoerythrin (PE) (clone 1 F11) and CD41-PE-cyanine 7 (CD41-PC7) (clone P2), as well as their respective isotype controls.

EMPs were enumerated by high-sensitivity flow cytometry following standardisation as previously described [41]. In brief, 30 μl of PFP was incubated with the appropriate amount of specific antibody or isotype control matched in terms of final concentration and fluorescence backgrounds, plus 10 μl of annexin V-FITC. Each stained sample was analysed on a NAVIOS-3 laser instrument (Beckman Coulter Life Sciences, Miami, FL, USA) following a protocol standardised with Megamix-Plus FSC beads (BioCytex, Marseille, France) with sizes ranging from 0.3 to 0.9 μm. EMPs were defined as annexin V^+^/CD31^+^CD41^−^ events. The absolute EMP counts (events per microlitre) were determined using ad hoc counting beads (CytoCount™; Dako, Glostrup, Denmark).

### Enumeration of circulating endothelial cells

CECs were isolated by immunomagnetic separation using Dynabeads coated with anti-CD146 monoclonal antibody (Life Technologies, Carlsbad, CA, USA) from 1 ml of blood samples collected into 5-ml ethylenediaminetetraacetic acid (EDTA) Vacutainer tubes. CECs were identified on the basis of previously described consensual morphologic and immunologic criteria [42]: rosette cell staining with acridine orange, size >15 μm, bearing more than five beads, and *Ulex europeaus* lectin-1 binding. Counting was performed with a fluorescence microscope.

### Assessment of colony-forming unit-endothelial cells

CFU-ECs were produced according to the protocol initially described by Hill et al. [25] and adapted by Smadja et al. [43]. Mononuclear cells (MNCs) were obtained by density gradient isolation and cultured with the EndoCult® Liquid Medium Kit (STEMCELL Technologies, Vancouver, BC, Canada) according to the manufacturer’s instructions. In short, MNCs were re-suspended in complete EndoCult® medium and seeded at 5 × 10^6^ cells/well in fibronectin-coated tissue culture plates (BD Biosciences, San Jose, CA, USA). After 48 h, to obtain CFU-ECs, non-adherent cells were collected and plated in EndoCult® buffer at 10^6^ cells/well in 24-well fibronectin-coated plates. CFU-EC colonies were counted after another 3 days, as recommended by the manufacturer.

### Enumeration and characterisation of circulating progenitor cells by flow cytometry

Blood samples were collected into 5-ml, EDTA-coated BD Vacutainer tubes. CD34^+^CD45^+^ haematopoietic progenitor cells (CD34^+^CD45^+^ HPCs) and CD34^+^CD45^−^ EPCs were enumerated with a whole-blood flow cytometry protocol adapted from the standardised International Society of Hematotherapy and Graft Engineering single-platform sequential gating strategy [44]. Briefly, 100 μl of blood was stained with FITC-CD45 antibody, PE-CD34 antibody or PE-immunoglobulin G1 (IgG1), and 7-aminoactinomycin D (7-AAD) (Stem-Kit Reagents; Beckman Coulter Life Sciences), according to the manufacturer’s instructions. After lysis of erythrocytes, flow count beads were added to each sample for absolute value determination, and samples were analysed with a NAVIOS flow cytometer equipped with CXP software (Beckman Coulter Life Sciences). At least 75,000 CD45^+^ cells were acquired per run. CD34^+^CD45^+^ HPCs and CD34^+^CD45^−^ EPCs were identified within 7-AAD-negative viable cells displaying forward scatter (FSC)/side scatter (SSC) characteristics corresponding to the lymphocyte cluster and CD45^dim^ or CD45^−^ expression. The results were expressed as absolute numbers of CD34^+^CD45^+^ HPCs and CD34^+^CD45^−^ EPCs per millilitre of blood. The gating strategy is illustrated in Additional file 2: Figure S1.

Because CD34^+^CD45^+^CD133^+^ PCs and CD34^+^CD45^+^KDR^+^ PCs, identified as CACs, are present in low levels in peripheral blood, these progenitor subsets were quantified using a four-color flow cytometry strategy after direct immunolabelling of isolated peripheral blood mononuclear cells (PBMCs). PBMCs were isolated from 7 ml of heparinised peripheral blood by density gradient centrifugation with lymphocyte separation medium (PAA Laboratories, Pasching, Austria), and they were labelled with 10 μl of 7-AAD viability dye, 10 μl of FITC-CD34 antibody (Beckman Coulter), 10 μl of ECD-CD45 antibody (Beckman Coulter) and 10 μl of PE-CD133 antibody (Miltenyi Biotec, Bergisch Gladbach, Germany) or PE-KDR antibody (R&D Systems, Abingdon, UK). A 10-μl quantity of concentration-matched, PE-conjugated murine IgG1 antibody was used as a fluorescence minus one control. After incubation for 20 minutes at room temperature, cells were washed and re-suspended in 500 μl of PBS, and then analysed using a NAVIOS flow cytometer.

After selection of 7-AAD-negative cells, CD133^+^ and KDR^+^ cells were identified within CD34^+^CD45^+^ cells displaying FSC/SSC characteristics corresponding to the lymphocyte cluster. At least 5 × 10^5^ viable cells were acquired per run. The percentage of CD133^+^ or KDR^+^ cells among CD34^+^CD45^+^ cells was determined. Results were expressed as absolute values per millilitre of blood of CD34^+^CD45^+^CD133^+^ PCs, and CD34^+^CD45^+^KDR^+^ PC values were obtained by multiplying the percentage of each cellular subpopulation by the absolute values of CD34^+^CD45^+^ HPCs determined as described above. The literature-based identification criteria and characteristics of the endothelial cell populations of the endothelial cell population investigated in the present study are summarised in the Additional file 1: Table S3.

### Analysis of circulating vascular endothelial growth factor, endothelin-1 and soluble fractalkine levels

Circulating levels of vascular endothelial growth factor (VEGF), endothelin-1 and s-Fractalkine (CX3CL1) were measured in serum using commercially available ELISA kits obtained from R&D Systems Inc. (Minneapolis, MN, USA). Assays were performed according to the manufacturer’s instructions.

### Statistical analysis

Quantitative variables were described using mean (±SD) or median and IQR [first quartile–third quartile] according to their distribution. The normality of the distribution was assessed graphically. Qualitative variables were described using counts and percentages. The correlations between quantitative variables were performed using Spearman’s correlation. Student’s *t* test and the Mann-Whitney *U* test were performed to compare using quantitative variables of the two groups. The chi-square test or Fisher’s exact test was used to compare qualitative variables. *P* values <0.05 were considered significant.

A multivariate model based on logistic regression with a robust estimator (generalised estimating equation with M-estimator) was used to assess independent factors associated with SSc. A stepwise backward elimination procedure was performed to conserve variables with an adjusted *P* value <0.05. Variables first introduced into the univariate model were those with a *P* value <0.20.

To analyse the association of the variables with clinical characteristics (cutaneous subtype, disease duration groups of severity of patients with SSc defined by Medsger severity scale), we first used quantitative values of these biomarkers. We next aimed to deepen the analysis in an exploratory prospect evaluation by segregating groups. We used decision trees with the chi-square automatic interaction detection (CHAID) method to assess whether cut-off values of these biomarker levels could enable us to discriminate these two groups of severity states in a dichotomous manner. Analyses were performed using Prism 5.0 (GraphPad Software, La Jolla, CA, USA) and IBM SPSS Statistics version 20.0 (IBM, Armonk, NY, USA) software.

## Results

### Characteristics of the study population

The characteristics of the study population, including cardiovascular risk factors and vascular phenotypes, are summarised in Table 1. SSc-related features are shown in Table 2, and the ongoing treatments are reported in Additional file 1: Table S1. The patient sample comprised 44 women and 1 man with a median age of 60.38 years [IQR 51.45–72.38 years] and median disease duration of 15 years. The control group of healthy volunteers consisted of 38 women and 3 men with a median age of 55.97 years [IQR 53.56–59.64 years]. Sixteen patients (35.6%) had diffuse cutaneous SSc, and 29 patients (64.4%) had limited cutaneous SSc. The demographic characteristics of patients with SSc were comparable to those of the healthy control subjects, except for higher mean age, lower body mass index (BMI) and lower arterial blood pressure observed in the patients with SSc (Table 1). The prevalence of cardiovascular risk factors, including diabetes, tobacco consumption, familial history of coronary disease, hypercholesterolemia, arterial hypertension, sedentary lifestyle, menarche, hormone treatment and atheroma deposits on carotid artery assessed by Doppler ultrasonography did not significantly differ between the two groups (data not shown). The right carotid-radial PWV did not significantly differ between the two groups, accounting for a similar arterial stiffness. However, patients with SSc had a significantly lower endothelium-dependent FMD of the brachial artery (Table 1).

Significant differences were observed between patients with SSc and control subjects regarding biological parameters such as white blood cell count, haemoglobin level and creatinine clearance. All of the values remained within the normal range. CRP level was slightly higher in the patient group than in healthy control subjects (Additional file 1: Table S2).

### Circulating cellular biomarkers of endothelial activation and injury in patients with SSc

**Fig. 1:**
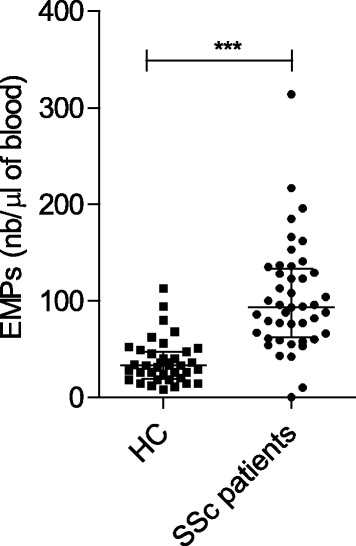
Quantification of endothelial microparticles (EMPs) in the peripheral blood of healthy control subjects (HCs) and patients with systemic sclerosis (SSc). EMP numbers (nb) were defined as annexin V^+^/CD31^+^CD41^−^ events and quantified by high-sensitivity flow cytometry using counting beads. *** *P* < 0.0005

Because EMPs and CECs reflect endothelial cell activation and injury, we aimed to investigate these markers in patients with SSc using standardised methods. The patients with SSc had significantly higher plasma levels of EMPs (93.5/μl, IQR 64.75–130.5) than control subjects (33/μl, IQR 21–46.5; *P* < 0.0001) (Fig. 1). Comparable levels of CECs were found in patients with SSc (2/ml of blood, IQR 1–8.5) and healthy control subjects (2.5/ml of blood, IQR 0–16.5).

### Colony forming unit-endothelial cell generation in patients with SSc

The assessment of CFU-ECs established by culture of MNCs from patients with SSc and healthy control subjects revealed higher numbers of colonies in patients with SSc (11 per 10^6^ MNCs, IQR 3–20.25) than in healthy control subjects (3 per 10^6^ MNCs, IQR 0.85–8; *P =* 0.0032) (Additional file 2: Figure S2).

### Flow cytometric analysis of circulating CD34^+^ progenitor cell subsets in patients with SSc

**Fig. 2:**
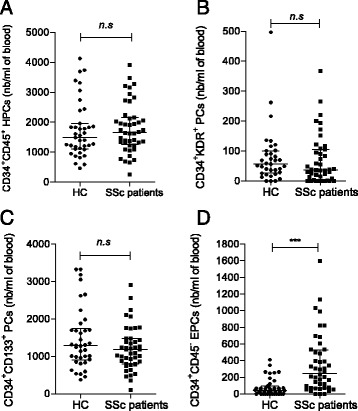
Circulating CD34^+^ progenitor cells (PCs) subsets counts in healthy control subjects (HC) and patients with systemic sclerosis (SSc). Number (nb) of (**a**) CD34^+^CD45^+^ haematopoietic progenitor cells (HPCs), (**b**) CD34^+^KDR^+^ PCs, (**c**) CD34^+^CD133^+^ PCs and (**d**) non-haematopoietic CD34^+^CD45^−^ endothelial PCs were determined by flow cytometric analysis. *** *P* < 0.0005. *n.s* Non-significant

To cover most of the literature-reported phenotypes of EPCs and CACs, we enumerated various circulating CD34^***+***^ cell populations, including total CD34^***+***^ PCs, CD34^***+***^CD45^−^ EPCs and HPCs, CD34^***+***^CD45^***+***^ HPCs, immature CD34^***+***^CD45^***+***^CD133^***+***^ PCs and CD34^***+***^CD45^***+***^KDR^***+***^ PCs. We observed that patients with SSc had significantly higher numbers of total circulating CD34^***+***^ PCs (2074.5/ml, IQR 1459.5–2578) than control subjects (1555/ml, IQR 1225–1960; *P* = 0.0442). The number of circulating HPCs did not differ significantly between patients with SSc and control subjects (1662/ml, IQR 1297.7–2134.2, versus 1485/ml, IQR 1117.5–1912.5; *P* = 0.4247) (Fig. 2a). Moreover, KDR^*+*^ PCs (37/ml, IQR 5.5–100.5, versus 57.01/ml, IQR 27.5–96; *P* = 0.243) and CD133^*+*^ PCs (1185/ml, IQR 827–1456, versus 1287/ml, IQR 929.71–1705.13; *P* = 0.2463) did not differ between the two groups (Fig. 2b and c). The number of circulating CD34^*+*^CD45^−^ EPCs was significantly higher in patients with SSc (247/ml, IQR 74.2–508.2, versus 40/ml, IQR 0–82.5; *P* <0.0001) (Fig. 2d). No correlation was found between EMPs (marker of endothelial activation) and PC subsets (CFU-ECs, CD34^***+***^ PCs and CD34^***+***^CD45^−^ EPCs) in patients with SSc.

### Elevation of soluble endothelial biomarkers in patients with SSc

**Fig. 3:**
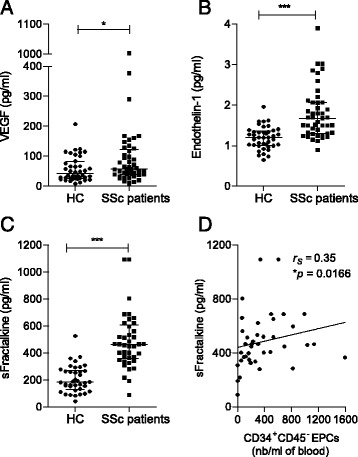
Soluble endothelial biomarker concentrations in patients with systemic sclerosis (SSc) compared with healthy control subjects (HC). **a** Vascular endothelial growth factor (VEGF). **b** Endothelin-1. **c** Soluble fractalkine (s-Fractalkine). **d** Correlation between s-Fractalkine and CD34^+^CD45^−^ endothelial progenitor cell (EPC) counts (nb). Correlation was established using the non-parametric Spearman’s correlation coefficient (*r*_*s*_). * *P* < 0.05; *** *P* < 0.0005. *n.s* = non-significant

First, we investigated the circulating levels of the endothelial biomarkers VEGF, s-Fractalkine and endothelin-1 in patients with SSc and healthy control subjects. Median serum concentrations of these biomarkers were significantly elevated in patients with SSc compared with control subjects: VEGF (57 pg/ml, IQR 39–118, versus 42 pg/ml, IQR 26–78.7; *P* = 0.0357) (Fig. 3a), endothelin-1 (1.68 pg/ml, IQR 1.3–2.0, versus 1.2 pg/ml, IQR 1.0–1.3; *P* < 0.0001) (Fig. 3b) and s-Fractalkine (465.45 pg/ml, IQR 366.4–598.5, versus 186 pg/ml, IQR 140.5–268.0 pg/ml; *P* < 0.0001) (Fig. 3c). Next, we assessed whether these soluble biomarkers correlated with the aforementioned cellular biomarkers in patients with SSc. We observed a significant positive correlation between s-Fractalkine levels and CD34^*+*^CD45^−^ EPC numbers (*r*_s_ = 0.35, *P* = 0.0166) (Fig. 3d). No other correlation was noted in patients with SSc.

### Association of endothelial biomarkers with SSc

The univariate analyses showed that the variables age, BMI, creatinine clearance and CRP, as well as the endothelial biomarkers EMPs, CFU-ECs, CD34^***+***^ PCs, CD34^***+***^CD45^−^ EPCs, VEGF, endothelin-1 and s-Fractalkine, were different between healthy control subjects and patients with SSc (*P* < 0.05) (Table 3).

**Table 3:** Circulating endothelial biomarkers studied in univariate analysis

	Patients with SSc	Control subjects	*P* value
Endothelial cellular markers			
EMPs, *n*/μl	93.5 [64.75–130.5]	33 [21– 46.5]	<0.0001***
CECs, *n*/ml	2 [1–8.5]	2.5 [0–16,5]	0.7677
PC subsets			
CFU-ECs, *n*/10^6^ MNCs	11 [3–20.25]	3 [0.85–8]	0.0032**
CD34^+^ PCs, *n*/ml	2074.5 [1459.5–2578]	1555 [1225–1960]	0.0442*
CD34^+^CD45^+^ PCs, *n*/ml	1662 [1297.7–2134.2]	1485 [1117.5–1912.5]	0.4247
KDR^+^ PCs, *n*/ml	37 [5.5–100.5]	57.01 [27.5–96]	0.243
CD133^+^ PCs, *n*/ml	1185 [827–1456]	1287 [929.71–1705.13]	0.2463
CD34^+^CD45^−^ EPCs, *n*/ml	247 [74.2–508.2]	40 [0–82.5]	<0.0001***
Soluble endothelial markers			
VEGF, pg/ml	57 [39–118]	42 [26–78.7]	0.0357*
Endothelin-1, pg/ml	1.68 [1.3–2.0]	1.2 [1.0–1.3]	<0.0001***
s-Fractalkine, pg/ml	465.45 [366.4–598.5]	186 [140.5–268.0]	<0.0001***

*Abbreviations: EMP* Endothelial microparticle, *CEC* Circulating endothelial cell, *CFU-EC* Colony-forming unit-endothelial cell, *EPC* Endothelial progenitor cell, *KDR* Kinas insert domain receptor, *PC* Progenitor cell, *VEGF* Vascular endothelial growth factor, *s-Fractalkine* Soluble fractalkine, *MNC* Mononuclear cell, *SSc* Systemic sclerosis * *P* < 0.05; 0.001 < **P < 0.01; *** *P* < 0.0005

In the multivariate model including all of these variables, EMPs, endothelin-1 and s-Fractalkine were independent markers associated with SSc (Table 4).

**Table 4:** Multivariate logistic regression analysis for systemic sclerosis with robust estimator

Variables	OR (95% CI)	*P* value
EMPs	1.002 (1.000-1.004)	0.015
Endothelin-1	1.044 (1.009-1.082)	0.012
s-Fractalkine	92.388 (2.729-3127.533)	0.035

*EMP* Endothelial microparticle, *s-Fractalkine* Soluble fractalkine

Variables introduced into this multivariate model were selected after univariate analysis (*P* < 0.2). The final model was established after stepwise backward elimination of non-significant variables (i.e., age, body mass index, creatinine, C-reactive protein, colony-forming unit-endothelial cells, CD34^***+***^ progenitor cells, CD34^***+***^CD45^−^ endothelial progenitor cells and vascular endothelial growth factor). Independent variables associated with systemic sclerosis with an adjusted *P* < 0.05 were conserved

Because s-Fractalkine level and CD34^*+*^CD45^−^ EPC count were correlated, a multivariate model with the same variables except for s-Fractalkine was tested. In this model, EMPs (*P* = 0.007), endothelin-1 (*P* = 0.001) and CD34^*+*^CD45^−^ EPCs (*P* = 0.025) were independently associated with SSc (Additional file 1: Table S4).

### Association of endothelial biomarkers with disease clinical characteristics and severity

We investigated whether each of the independent biomarkers associated with SSc (i.e., EMPs, CD34^+^CD45^−^ EPCs, endothelin-1 and s-Fractalkine) could be associated with the clinical characteristics of patients with SSc. The first level of analysis of the quantitative values of these biomarkers did not allow for identifying markers that discriminated the cutaneous subtype, disease duration, clinical characteristic and group of severity defined by the Medsger severity scale (*P* > 0.05 for all).

The CHAID algorithm allowed us to propose cut-off values for s-Fractalkine (486 pg/ml), CD34^+^CD45^−^ EPC level (395/ml) and endothelin-1 (1.26 ng/ml). According to this approach, values of EMPs could not be discriminated in a dichotomous manner.

The frequency of patients with more severe disease was higher in the group with s-Fractalkine concentrations above the cut-off value (*P* = 0.041). Patients belonging to this group also had decreased DLCO/VA and a higher frequency of pulmonary fibrosis (Table 5). Patients with lower FVC had CD34^+^CD45^−^ EPC numbers above the cut-off value (*P* = 0.0282). No significant difference was noted between patients with endothelin-1 levels less than or greater than the cut-off value.

**Table 5:** Clinical and laboratory data of patients with systemic sclerosis, depending on soluble fractalkine concentrations

	s-Fractalkine >486 pg/ml (*n* = 16)	s-Fractalkine <486 pg/ml (*n* = 25)	*P* value
Clinical features			
Age, years, median [IQR]	55.06 [50.94–72.84]	62.52 [52.8–71.33]	0.56
Duration, years, median [IQR]	5.5 [3–12.75]	8 [3–12.5]	0.8933
Pitting scars or skin ulcers, *n*	11/16	14/25	0.41
Pulmonary fibrosis, *n*	3/16	3/22	<0.0001***
FVC, %, median [IQR]	98.5[85.75–113.3]	95.9 [82–111.3]	0.558
DLCO/VA, %, median [IQR]	62 [53–76]	75 [63.5–81.45]	0.0445*
Oesophagus, *n*	13/16	13/25	0.0579
Telangiectasia, *n*	9/16	20/25	0.1030
mRSS, median [IQR]	11.5 [5.25–23]	10 [5–16]	0.6166
Pulmonary arterial pressure, mmHg, median [IQR]	27 [21.25–34]	25 [20–31]	0.32
Severity grades 0, 1 and 2, *n*	4/16	15/25	0.0284*
Severity grades 3 and 4, *n*	12/16	10/25	0.0284*
Laboratory findings			
Anti-topoisomerase I antibody, *n*	6/16	13/25	0.3638
Anti-centromere antibody, *n*	7/16	12/25	0.79

*Abbreviations: DLCO* Diffusing capacity of the lung for carbon monoxide, *CEC* Circulating endothelial cell, *FVC* Forced vital capacity, *mRSS* Modified Rodnan skin score, *VA* Alveolar volume, *s-Fractalkine* Soluble fractalkine

Qualitative variables are described using frequencies. Quantitative variables are described using median and IQR [first quartile–third quartile] * *P* < 0.05; *** *P* < 0.0005

## Discussion

The non-invasive monitoring of endothelial alterations is a challenge for physicians to better assess the severity of the disease and to sustain the development of therapeutic approaches [45, 46]. Based on an integrative analysis of endothelial biomarkers of damage and repair, this study identifies the mobilisation of CD34^+^CD45^−^ EPCs and high levels of s-Fractalkine as specific features of SSc-associated vascular activation and disease severity.

We confirmed the impaired endothelial function assessed by FMD in patients with SSc [2]. Consistent with a disrupted endothelial homeostasis [9], we confirmed that EMP release is increased in patients with SSc. The EMPs can promote or aggravate pre-existing vascular dysfunction in cardiovascular diseases [4] and have been shown to be deleterious via the induction of an oxidative burst in a murine model of SSc [47]. In the present study, EMP levels were not associated with disease severity. Researchers in other studies reported decreased levels of EMPs in SSc [48] or their association with lung and skin involvement, but they did not consider annexin V binding as a criterion to define EMPs [49]. Our observations indicate that the release of annexin V^+^ EMPs primarily reflects the activated endothelial status. Such activation might not result in a significant insult of the endothelial monolayer, given that the release of EMPs was not associated with CEC elevation assessed by anti-CD146-based immunomagnetic separation. Indeed, this standardised assay has been shown to quantify mature endothelial cells that are detached from the endothelial monolayer as a consequence of pathological processes that disrupt its integrity [6]. Such a restricted definition criterion of CECs might explain discrepancies observed in other studies in which researchers used flow cytometric approaches to quantify CECs in patients with SSc [14].

We also present the first evidence that enhanced levels of circulating CD34^+^CD45^−^ EPCs occur in patients with SSc, whereas these cells were barely detectable in healthy control subjects. The association between CD34^+^CD45^−^ EPC levels and decreased FVC may suggest that these cells are mobilised as a response to lung involvement-induced hypoxia. In accordance with this hypothesis, patients with SSc exhibited high levels of VEGF, the main pro-angiogenic factor promoting the mobilisation of PCs from the bone marrow [11]. Interestingly, CD34^+^CD45^−^ EPC counts were increased, whereas all the haematopoietic lineage-related CD34^+^ PC subsets defined by CD45^dim^ expression and CD133 or KDR markers remained unchanged in the analysed cohort. These data may indicate defective mobilisation of the bone marrow-derived pool of these CACs, as reported by Kuwana et al. [13]*.* In line with this hypothesis, quantitative and qualitative alterations of this bone marrow pool of HPCs have been described in SSc [17]. Of note, our flow cytometric analysis did not include the circulating cells co-expressing CD133, CD34 and KDR. Although this phenotype was initially proposed to identify the immature part of EPC populations, we failed to detect these cells in both healthy control subjects and patients with SSc (data not shown), as recently published [50].

The functional ability of the cells derived from mobilised CD34^+^CD45^−^ EPCs could not be analysed in the present study, because the clonogenic and angiogenic assays require large blood samples that are hardly obtainable from patients with SSc. Nevertheless, Avouac et al. observed that the number of late-outgrowth ECFCs derived in culture was not increased in patients with SSc in comparison with control subjects [16]. Along with our results, we can assume that the mobilisation of CD34^+^CD45^−^ EPCs may represent a quantitative response to activation that is not translated in the acquisition of progenitor-associated vascular regenerative potential.

In this study, the mobilisation of CD34^+^CD45^−^ EPCs occurred in a context of vascular inflammation, as revealed by the moderate elevation of CRP and CFU-ECs and the increase in endothelial activation biomarkers such as EMPs, endothelin-1 and s-Fractalkine. Furthermore, we established a positive correlation between the CD34^+^CD45^−^ EPC levels and the circulating levels of s-Fractalkine in patients with SSc. Animal models suggest that the fractalkine/CX3CR1 pathway is involved in the recruitment of EPCs to the ischaemic sites during stroke [51] and regulates progenitor-dependent endothelial repair during atherosclerosis [52]. Moreover, we have previously shown that membrane-bound fractalkine can play a role in controlling EPC homeostasis in the alloreactive environment of renal transplant [53]. The first evidence provided here of the interplay between s-Fractalkine and EPCs in the context of SSc may deserve further mechanistic investigation. In the present study, the SSc group exhibited reduced FMD values but similar arterial stiffness in comparison with the control group, suggesting that the alterations of the biological markers observed here were due to the SSc-related endothelial dysfunction rather than to atherosclerosis.

The limits of our exploratory study are inherent to a one-point biological assessment performed in a low number of patients with heterogeneous clinical phenotypes. Furthermore, the impact of long-standing therapies could not be appreciated. This could also account in part for the lack of association between endothelin-1 levels and disease severity, notably in patients treated with endothelin-1 receptor antagonists. s-Fractalkine was the only soluble marker found to be associated with disease severity in SSc in this study [30]. Such an observation is consistent with the involvement of the fractalkine/CX3CR1 pathway in the mechanisms linking the endothelial inflammation to the onset of fibrosis.

## Conclusions

Our data identify a specific signature of inflammatory endothelial activation that encompasses high levels of EMPs, CFU-ECs, endothelin-1, VEGF and s-Fractalkine and links the levels of s-Fractalkine with the mobilisation of CD34^+^CD45^−^ EPCs. s-Fractalkine and CD34^+^CD45^−^ EPCs seem to be associated with lung involvement. Further studies are needed to determine the exact prognostic value of the combined assessment of s-Fractalkine and CD34^+^CD45^−^ EPCs in patients with SSc.

## Additional files

Additional file 1: Table S1. Ongoing treatments in patients with SSc. **Table S2.** Biological characteristics of the study population. **Table S3.** Summary of the main immunophenotypic and functional characteristics of the investigated circulating progenitor cell subsets. **Table S4.** Multivariate logistic regression analysis for SSc with robust estimator. (DOCX 16 kb)
Additional file 2: Figure S1. Gating strategy of endothelial progenitor cells (EPCs) by flow cytometry. CD34^+^CD45^+^ haematopoietic progenitor cells (HPCs) and CD34^+^CD45^−^ EPCs were identified within 7-AAD-negative viable (**a**), CD34^+^ cells (**b**), with CD45^dim^ or CD45^−^ expression (**c**), and displaying forward scatter (FS)/side scatter (SS) characteristics corresponding to the lymphocyte cluster (**d**, **e**). Non-specific binding of CD34-PE antibody is checked on CD34^+^CD45^+^ (**f**) and CD34^+^CD45^−^ gate of each sample (**g**) by means of a control tube in which CD34-PE antibody is replaced by its isoclonic control. *LYMPH* Lymphocytes. **Figure S2.** CFU-EC count in the peripheral venous blood of healthy control subjects (HC) and patients with systemic sclerosis (SSc). Number (n) of CFU-ECs was determined after cell culture of MNCs. ** *P* < 0.005. (PDF 311 kb)

## Abbreviations

7-AAD: 7-Aminoactinomycin D
APHM: Assistance Publique-Hôpitaux de Marseille
BMI: Body mass index
CAC: Circulating angiogenic cell
CEC: Circulating endothelial cell
CFU-EC: Colony-forming unit-endothelial cell
CHAID: Chi-square automatic interaction detection
CRP: C-reactive protein
CX3CL1: Chemokine (C-X3-C motif) ligand 1
CX3CR1: Chemokine (C-X3-C motif) receptor 1
DLCO: Diffusing capacity of the lung for carbon monoxide
ECFC: Endothelial colony-forming cell
EDTA: Ethylenediaminetetraacetic acid
ELISA: Enzyme-linked immunosorbent assay
EMP: Endothelial microparticle
EPC: Endothelial progenitor cell
FITC: Fluorescein isothiocyanate
FMD: Flow-mediated dilation
FSC: Forward scatter
FVC: Forced vital capacity
HC: Healthy control subject
HDL: High-density lipoprotein
HPC: Haematopoietic progenitor cell
IgG1: Immunoglobulin G1
KDR: Kinase insert domain receptor
LDL: Low-density lipoprotein
MNC: Mononuclear cell
mRSS: Modified Rodnan skin score
PBMC: Peripheral blood mononuclear cell
PC: Progenitor cell
PE: Phycoerythrin
PFP: Platelet-free plasma
PWV: Pulse wave velocity
s-Fractalkine: Soluble fractalkine
SSC: Side scatter
SSc: Systemic sclerosis
VA: alveolar volume
VEGF: Vascular endothelial growth factor
