# Supplementary material for: Increased serum levels of fractalkine and mobilisation of CD34+CD45− endothelial progenitor cells in systemic sclerosis
Source: Arthritis Res Ther. 2017 Mar 20;19:60. doi: 10.1186/s13075-017-1271-7 (PMC5359964; doi:10.1186/s13075-017-1271-7)
Supplement: Additional file 1: Table S1. — Ongoing treatments in patients with SSc. Table S2. Biological characteristics of the study population. Table S3. Summary of the main immunophenotypic and functional characteristics of the investigated circulating progenitor cell subsets. Table S4. Multivariate logistic regression analysis for SSc with robust estimator. (DOCX 16 kb) [file 13075_2017_1271_MOESM1_ESM.docx]

**Additional File 1: Supplementary Tables (Table S1-4)**

**Table S1: Ongoing treatments in SSc patients**

| **Treatments** | **Number of SSc patients (%)** |
| --- | --- |
| Bosentan | 9 (20.0%) |
| Iloprost | 5 (11.1%) |
| Corticosteroid | 1 (2.2%) |
| Methotrexate | 3 (6.7%) |
| Mycophenolate Mofetil | 3 (6.7%) |
| Azathioprine | 1 (2.2%) |
| Calcium-channel blocker | 20 (7.1%) |
| Angiotensin-converting enzyme inhibitor or Angiotensin II Receptor Blocker | 7 (15.6%) |

**Table S2: Biological characteristics of the study population.**

|  | SSc Patients | Controls | p-value |
| --- | --- | --- | --- |
| Haematocrit (%) | 39 [ 38 - 41] | 40 [ 38 - 41] | 0.378 |
| Haemoglobin (g/dl) | 128 [ 122 - 133] | 132 [ 127 - 139] | 0.0036 |
| Red cell count (Tera/l) | 4.41 [ 4.26 - 4.71] | 4.54 [ 4.3 - 4.72] | 0.511 |
| White Blood Cell count (Giga/l) | 6.9 [ 6 - 8.15] | 5.79 [ 4.91 - 7.15] | 0.003 |
| Neutrophils (Giga/l) | 4.33 [ 3.48 - 5.4] | 3.2 [ 2.64 - 4.1] | 0.0007 |
| Eosinophils (Giga/l) | 0.17 [ 0.12 - 0.24] | 0.15 [ 0.11 - 0.22] | 0.5051 |
| Lymphocytes (Giga/l) | 1.92 [ 1.5 - 2.18] | 1.79 [ 1.53 - 2.3] | 0.9862 |
| Monocytes (Giga/l) | 0.45 [ 0.34 - 0.56] | 0.31 [ 0.24 - 0.38] | 0.0004 |
| Platelets (Giga/l) | 318 [ 277 - 344] | 275 [ 249.75 - 330] | 0.0522 |
| Creatinine (µmol/l) | 64 [ 58.9 - 72] | 64 [ 55 - 69] | 0.5053 |
| Creatinine clearance (ml/min) | 72 [ 60 - 87] | 92 [ 79 - 105] | 0.0005 |
| C Reactive Protein (mg/l) | 5 [ 5 – 12] | 5 [ 5 - 5] | 0.0205 |

Variables are described using median [1st quartile – 3rd quartile].

**Table S3: Summary of the main immunophenotypic and functional characteristics of the investigated circulating progenitor cell subsets.**

| **Cell subset** | **Phenotype/identification criteria** | **characteristics/function** |
| --- | --- | --- |
| **CD34+ Progenitor cell subsets determined by flow cytometry** | | |
| Haematopoietic progenitor cells | CD34^+^CD45^+^ | Cells related to the haematopoietic lineage,  Circulating Angiogenic Cells able to support angiogenesis through paracrine mechanisms |
| Circulating progenitor cells expressing immaturity marker | CD34^+^CD45^+^CD133^+^ |  |
| Circulating progenitor cells expressing endothelial marker | CD34^+^CD45^+^KDR^+^ |  |
| Endothelial progenitor cells | CD34^+^CD45^-^ | Progenitor cells unrelated to the haematopoietic lineage with a specific ability for endothelial differentiation and *de novo* vessel formation |
| **Cell subset detected using *ex vivo* culture assay** | | |
| Colony-Forming Units-Endothelial cells (CFU-ECs) | Blood-derived fibronectin adherent mononuclear cells | Circulating cells displaying mainly monocytes/macrophages characteristics,  Cells with a paracrine supportive function in angiogenesis and endothelial repair |

**Table S4: Multivariate logistic regression analysis for SSc with robust estimator**

| **Variables** | **OR (95 % CI)** | ***P-*value** |
| --- | --- | --- |
| EMPs | 1.064 (1.017-1.113) | 0.007 |
| Endothelin-1 | 165.505 (7.308-3748.084) | 0.001 |
| CD34^+^CD45^-^ EPC | 1.004 (1.001-1.008) | 0.025 |

Variables introduced in this multivariate model were selected after univariate analysis (*P*<0.2) and the variable sFractalkine was excluded. The final model was established after stepwise backward elimination of non-significant variables. The final model was established after stepwise backward elimination of non-significant variables *i.e.* age, BMI, creatinine, CRP, CFU-ECs, CD34***^+^*** PCs and VEGF. Were conserved independent variables associated with SSc disease with an adjusted *P*<0.05. OR= odds ratio. 95% CI= 95% confidence interval.
