# Supplementary figures and images for: Increased serum levels of fractalkine and mobilisation of CD34+CD45− endothelial progenitor cells in systemic sclerosis
Source: Arthritis Res Ther. 2017 Mar 20;19:60. doi: 10.1186/s13075-017-1271-7 (PMC5359964; doi:10.1186/s13075-017-1271-7)

Figure S1

A

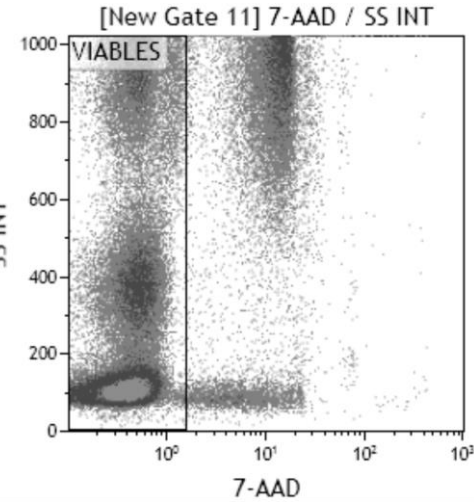

B

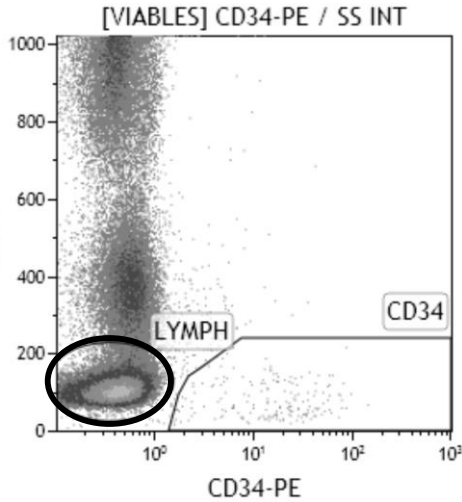

C

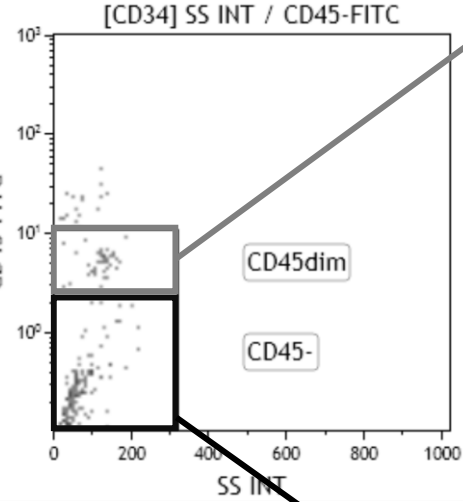

D

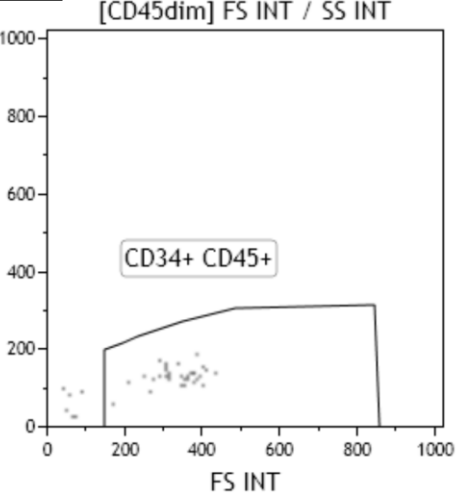

F

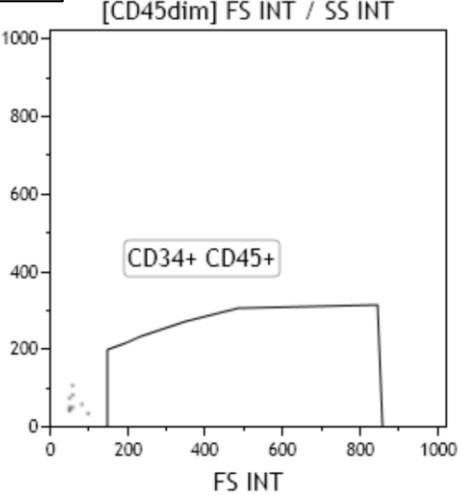

E

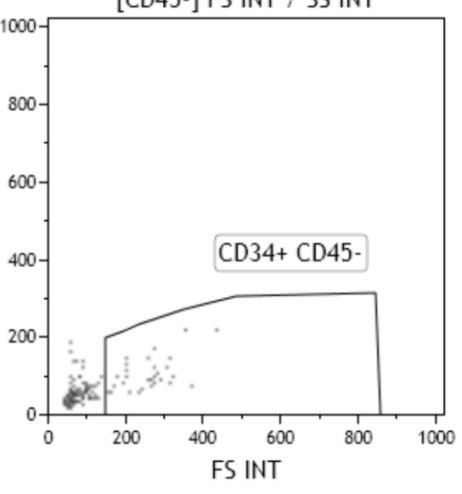

G

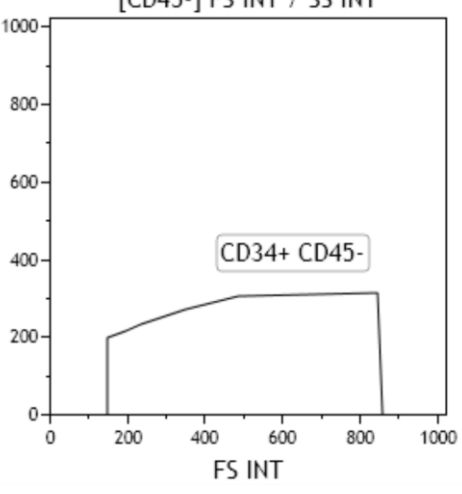

## Figure S2

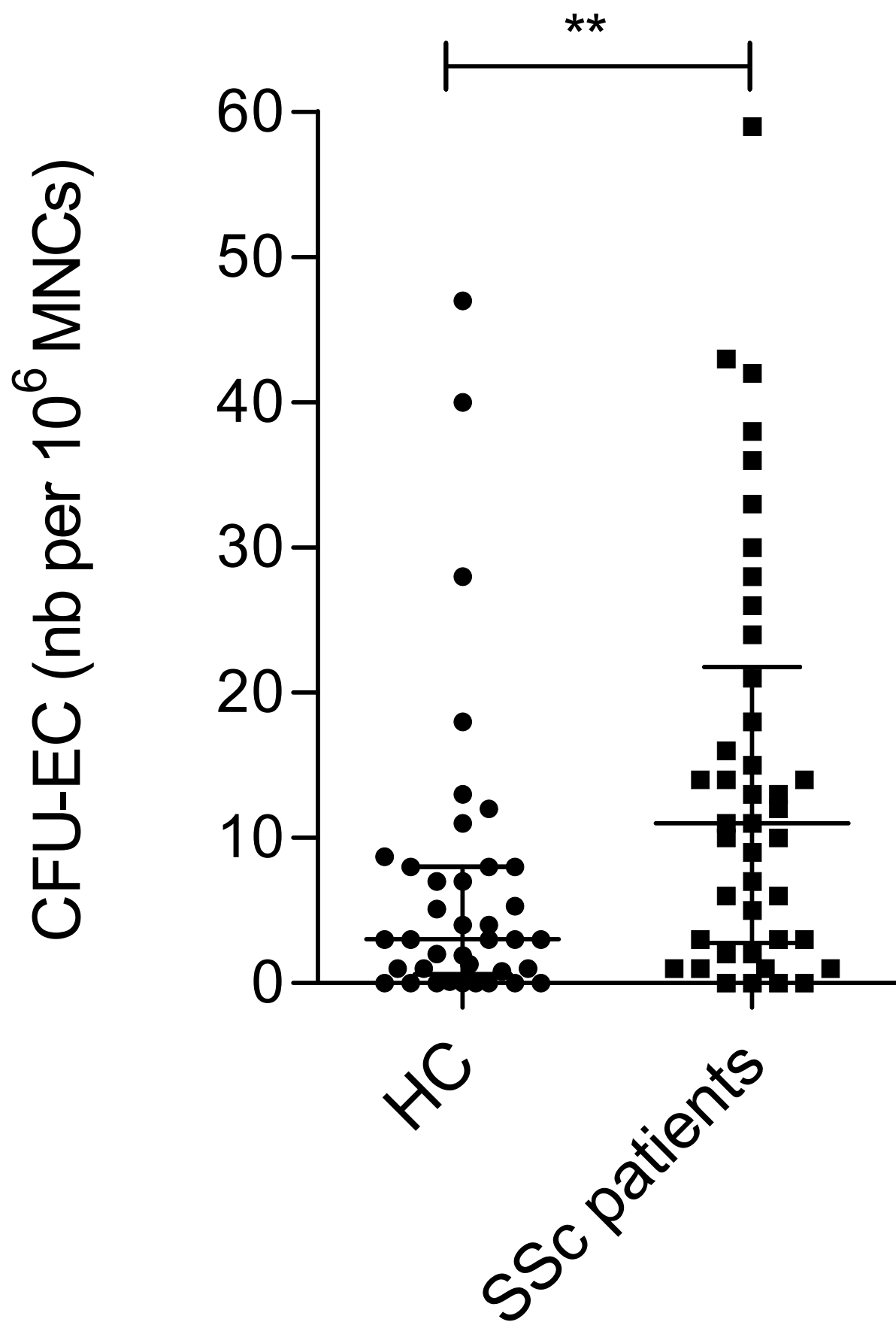

Supplement: Additional file 2: Figure S1. — Gating strategy of endothelial progenitor cells (EPCs) by flow cytometry. CD34+CD45+ haematopoietic progenitor cells (HPCs) and CD34+CD45− EPCs were identified within 7-AAD-negative viable (a), CD34+ cells (b), with CD45dim or CD45− expression (c), and displaying forward scatter (FS)/side scatter (SS) characteristics corresponding to the lymphocyte cluster (d, e). Non-specific binding of CD34-PE antibody is checked on CD34+CD45+ (f) and CD34+CD45− gate of each sample (g) by means of a control tube in which CD34-PE antibody is replaced by its isoclonic control. LYMPH Lymphocytes. Figure S2. CFU-EC count in the peripheral venous blood of healthy control subjects (HC) and patients with systemic sclerosis (SSc). Number (n) of CFU-ECs was determined after cell culture of MNCs. ** P < 0.005. (PDF 311 kb) [file 13075_2017_1271_MOESM2_ESM.pdf]
